# Spatiotemporal pattern and suitable areas analysis of equine influenza in global scale (2005–2022)

**DOI:** 10.3389/fvets.2024.1395327

**Published:** 2024-06-03

**Authors:** Jiafeng Ding, Yu Wang, Jinjiao Liang, Zhenhuan He, Changhong Zhai, Yinghao He, Jiayin Xu, Lei Lei, Jing Mu, Min Zheng, Boyang Liu, Mingxian Shi

**Affiliations:** ^1^College of Animal Science and Technology, Guangxi University, Nanning, China; ^2^Guangxi Key Laboratory of Animal Breeding, Disease Control and Prevention, Guangxi University, Nanning, China; ^3^Nanning New Technology Entrepreneur Center, Nanning, China; ^4^Shenyang Zhengda Animal Husbandry Co., Ltd., Shenyang, China; ^5^Guangxi Center for Animal Disease Control and Prevention, Nanning, China; ^6^College of Wildlife and Protected Area, Northeast Forestry University, Harbin, China

**Keywords:** equine influenza, spatial epidemiology, spatiotemporal pattern, suitable area, Maxent model

## Abstract

Equine influenza (EI) is a severe infectious disease that causes huge economic losses to the horse industry. Spatial epidemiology technology can explore the spatiotemporal distribution characteristics and occurrence risks of infectious diseases, it has played an important role in the prevention and control of major infectious diseases in humans and animals. For the first time, this study conducted a systematic analysis of the spatiotemporal distribution of EI using SaTScan software and investigated the important environmental variables and suitable areas for EI occurrence using the Maxent model. A total of 517 occurrences of EI from 2005 to 2022 were evaluated, and 14 significant spatiotemporal clusters were identified. Furthermore, a Maxent model was successfully established with high prediction accuracy (AUC = 0.920 ± 0.008). The results indicated that annual average ultraviolet radiation, horse density, and precipitation of the coldest quarter were the three most important environmental variables affecting EI occurrence. The suitable areas for EI occurrence are widely distributed across all continents, especially in Asia (India, Mongolia, and China) and the Americas (Brazil, Uruguay, USA, and Mexico). In the future, these suitable areas will expand and move eastward. The largest expansion is predicted under SSP126 scenarios, while the opposite trend will be observed under SSP585 scenarios. This study presents the spatial epidemiological characteristics of EI for the first time. The results could provide valuable scientific insights that can effectively inform prevention and control strategies in regions at risk of EI worldwide.

## 1 Introduction

Equine influenza (EI) is a highly contagious respiratory disease caused by the equine influenza virus (EIV) (1). This disease can cause significant economic losses to the global equine industry (2). Clinical symptoms of EI include fever, dry cough, and runny nose (3). EI is primarily transmitted through aerosols from infected horses and can spread quickly within a group of horses and to other susceptible animals nearby (4). EIV is classified into two subtypes, H7N7 and H3N8 (5). The H3N8 subtype is currently the predominant epidemic type worldwide, including both European and American lineages (6). The American lineages are further categorized into South American, Kentucky, and Florida lineages, with the Florida lineages having two additional branches. Florida Clade 1 is primarily found in the Americas and Europe, whereas Florida Clade 2 is primarily found in Europe, Asia, and Africa. Vaccination is currently considered the primary means of preventing EI, but it actually has not achieved the desired expectation (7).

Space epidemiology is an effective tool to study the risk and influencing factors of infectious disease transmission (8). The use of spatiotemporal analysis and niche models (ENMs) has focused on establishing prevention strategies for infectious diseases. Spatiotemporal analysis is a technique used to study the spatiotemporal distribution characteristics of infectious diseases, which can accurately predict the spatiotemporal distribution (clusters) of human and animal infectious diseases (9). Wong et al. conducted a study using spatiotemporal cluster analysis to investigate the geographical variation of syphilis epidemiology and predict possible outbreaks in south China (10). Their results suggest that spatiotemporal cluster analysis could be used to focus provincial syphilis control programs. Gao et al. successfully utilized spatiotemporal cluster analysis to identify the spatial distribution and risk areas of foot and mouth disease (FMD) in mainland China, providing valuable insights for decision-makers to tailor a risk-based surveillance of FMD in China (11). It is worth noting that, to date, no literature has explored the spatiotemporal distribution characteristics of EI using spatiotemporal cluster analysis.

ENMs link environmental variables to disease occurrence, elucidate the environmental conditions required for the survival and spread of infectious disease pathogens, identify environmental similarities between the study area and known areas of pathogen distribution, and understand the ecological characteristics and geographic distribution of pathogens (12). The Maxent model is a widely used ENMs that accurately predicts suitable areas with limited generation data, demonstrating excellent performance (13). Ren et al. used Maxent models to successfully identify potential risk areas for coronavirus disease 2019 in its early stages (14). Their findings were instrumental in implementing targeted prevention and control strategies. Similarly, Choi et al. utilized the Maxent model to accurately predict suitable areas for African swine fever (ASF) outbreaks in wild boars in South Korea (15). Their findings established preemptive measures to prevent ASF in pig farming sectors at risk of ASF spillover from wild boars. To date, no literature has analyzed the environmental variables and suitable areas for EI occurrence via Maxent model.

EI has become widespread worldwide in recent years, and existing studies have proven that environmental variables such as temperature and humidity have an important impact on the occurrence and spread of EI (16). Bioclimatic variables can faithfully reflect monthly temperature and precipitation values, and have been widely used in the prediction of various infectious diseases (17). In this study, the current and future bioclimatic variables were selected and combined with EI occurrence data, and the Maxent model was constructed to analyze the relationship between EI occurrence and climate change.

This study aims to apply spatiotemporal cluster analysis and the Maxent model to not only map the distribution of EI but also identify key environmental drivers and predict future outbreaks. The results could provide valuable scientific insights that can inform prevention and control strategies in regions at risk of EI worldwide.

## 2 Materials and methods

### 2.1 Data collection and processing

**Figure 1:**
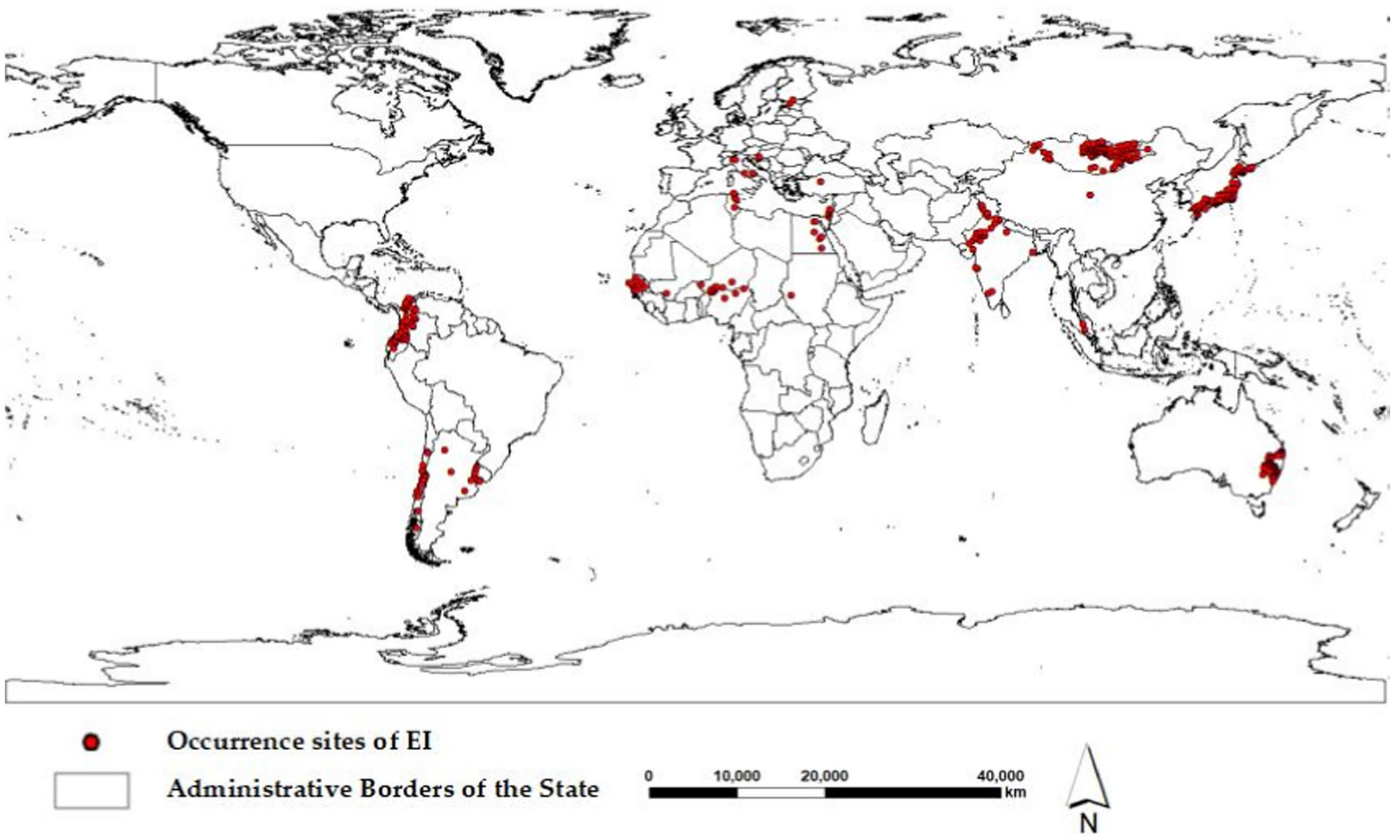
The 517 global occurrence sites of equine influenza (EI) from 2005 to 2022.

A comprehensive dataset of 696 global occurrence of the H3N8 subtype of EI between January 1, 2005, and December 31, 2022, was carefully collected from the Global Animal Disease Information System of the Food and Agriculture Organization of the United Nations (FAO, https://empres-i.apps.fao.org). The dataset includes the precise latitude and longitude coordinates of the occurrence, the occurrence time, and the number of affected animals. To ensure the accuracy of the Maxent model, the occurrence data were purified with ENMTools to remove the influence of spatial autocorrelation (18). Finally, the Maxent model was trained using 517 global occurrence data points of EI, as shown in Figure 1.

Twenty-one environmental variables were considered to build the Maxent model for EI, including 19 bioclimatic variables (bio_1–19), as well as 2 related variables, including annual average ultraviolet radiation (uvb1) and horse density (hd). The 19 bioclimatic variables, representing global climate conditions from 1970 to 2000, were obtained from the widely-used Worldclim 2.0 database^1^ (19). The uvb1 data were downloaded from the website of the Helmholtz Center for Environmental Research.^2^ Data for hd were obtained from the Global Livestock Distribution Database on the FAO website.^3^ The Maxent model used the twenty-one environmental variables listed in Table 1.

**Table 1:** The twenty-one environmental variables used in the Maxent model.

Variables	Description	Unit
bio_1	Annual mean temperature	°C
bio_2	Mean diurnal range	°C
bio_3	Isothermality	/
bio_4	Temperature seasonality	°C
bio_5	Max temperature of warmest month	°C
bio_6	Min temperature of coldest month	°C
bio_7	Temperature annual range	°C
bio_8	Mean temperature of wettest quarter	°C
bio_9	Mean temperature of driest quarter	°C
bio_10	Mean temperature of warmest quarter	°C
bio_11	Mean temperature of coldest quarter	°C
bio_12	Annual precipitation	mm
bio_13	Precipitation of wettest month	mm
bio_14	Precipitation of driest month	mm
bio_15	Precipitation seasonality	/
bio_16	Precipitation of wettest quarter	mm
bio_17	Precipitation of driest quarter	mm
bio_18	Precipitation of warmest quarter	mm
bio_19	Precipitation of coldest quarter	mm
uvb1	Annual mean ultraviolet radiation	J/m^2^/day
hd	Horse density	Bolt/km^2^

The data for future climate scenarios was obtained from the Worldclim 2.0 database using the sixth coupled model (BCC-CSM2-MR) of the Coupled Model Intercomparison Project (CMIP6), compared with the CMIP5 model, the CMIP6 model improves the simulation ability of the current regional temperature and precipitation in China, and the precipitation index is significantly improved (20). This model simulates the response of global climate to increased greenhouse gas. The Shared Socio-economic Pathways (SSP) consider a range of factors that affect future greenhouse gas emissions, including economic factors, population, economic growth, urbanization, and other socio-economic factors (21). This pathway provides a comprehensive view of future climate change from a long-term perspective within a predetermined scenario. For years 2050 (2041–2060 average) and years 2070 (2061–2080 average), three widely-used socioeconomic pathways were selected: SSP126 (represents the lowest emissions scenario, which is a sustainability-focused pathway), SSP245 (represents a medium emissions scenario and is a pathway where the trend largely follows its historical pattern), and SSP585 (represents the highest emissions scenario, which is a pathway dominated by traditional fossil fuels). We resolved the current and future environmental variables at a 2.5 arcminute resolution, corresponding to 21.6 square kilometers per grid.

### 2.2 Spatiotemporal cluster analysis

Spatiotemporal cluster analysis was conducted using SaTScan version 10.1 to explore the distribution characteristics of EI occurrence. In SaTScan, the spatiotemporal rearrangement scanning statistics model was selected to identify potential spatiotemporal clusters by forming a dynamic scanning window of occurrence data, which includes the number and location of onset cases, the spatiotemporal scanning statistical model can better detect the spatiotemporal aggregation patterns of infectious diseases (9). The clustering of scanning windows was evaluated using the generalized likelihood ratio (GLR). The study set a maximum spatial cluster size of 1,000 km and a maximum temporal cluster size of 20%. The *p* value was calculated using Monte Carlo random sampling to generate the simulation dataset (22). Any spatiotemporal cluster with a *p* value <0.05 was considered statistically significant. SaTScan analyzed the dataset of significant spatiotemporal clusters, which were then visualized using ArcGIS software version 10.2.

### 2.3 Maxent modeling

**Figure 2:**
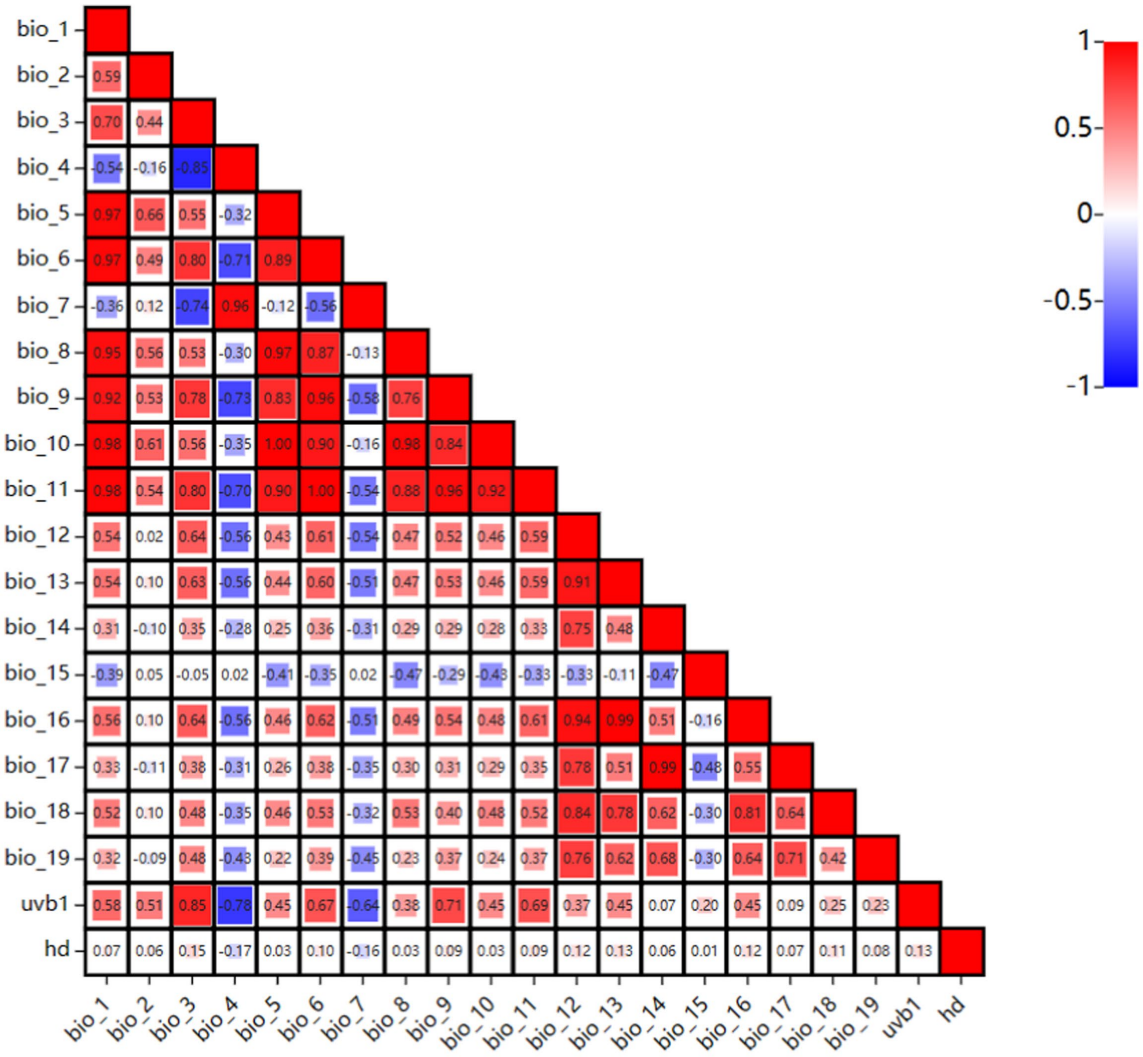
The visualization matrix of correlation coefficients for the twenty-one environmental variables.

To predict the global suitable areas for EI occurrence, a Maxent model was built, using Maxent software version 3.4.1. To ensure the model’s accuracy, ENMTools was employed to test the correlation of bioclimatic variables (23). The visualization matrix of correlation coefficients for the twenty-one environmental variables was shown in Figure 2. The figure displays stronger correlations with darker red and darker blue. A correlation was considered strong when the correlation coefficient between each pair of variables |r| > 0.7 (24). Strong correlations between variables can lead to overfitting, which can affect modeling results. Therefore, the contribution rate of each variable was determined using Maxent software, and eliminate the variable with a smaller contribution rate among the relevant variables. The final model includes eight variables: bio_2, bio_3, bio_5, bio_13, bio_15, bio_19, hd, and uvb1. Eight environmental variables and the data of 517 occurrence were imported into the Maxent model. In the Maxent software, set Random Test Percentage to 25, which means that 25% of the data will be randomly selected as a test set to test the model. Set Replicates to 10, which means the model was run 10 times and the average was used as the final result. Regularization Multiplier was set to 1, and Replicate Run Type was subsampled (the formula was substituted into the model for operation, and the mode with the best final result was selected). The maximum number of background points was set to 10,000, and other settings were left as default.

The Maxent model’s performance was evaluated by calculating the area under the curve (AUC) of the ROC curve (25). A higher AUC value indicates better predictive performance, with the prediction considered failed (0.5–0.6), poor (0.6–0.7), fair (0.7–0.8), good (0.8–0.9), or excellent (0.9–1). The corresponding response curve was used to determine the impact of these variables on the suitable areas of EI occurrence (26). Moreover, the importance of the eight environmental variables in the Maxent model was evaluated, respectively, using the jackknife test (27). The probability of occurrence in suitable areas ranges from 0 to 1, with a higher value indicating a greater probability. Based on the obtained results, the suitable areas can be further divided into four levels using maximum test sensitivity plus specificity (MTSPS): unsuitable (0–MTSPS), lowly suitable (MTSPS–0.5), mediumly suitable (0.5–0.6), and highly suitable (0.6–1) (28). The sizes and locations of suitable areas for the current and future scenarios were compared using ArcGIS version 10.2. Centroid position shifts of the suitable areas were also calculated.

## 3 Results

### 3.1 Spatiotemporal cluster analysis

**Figure 3:**
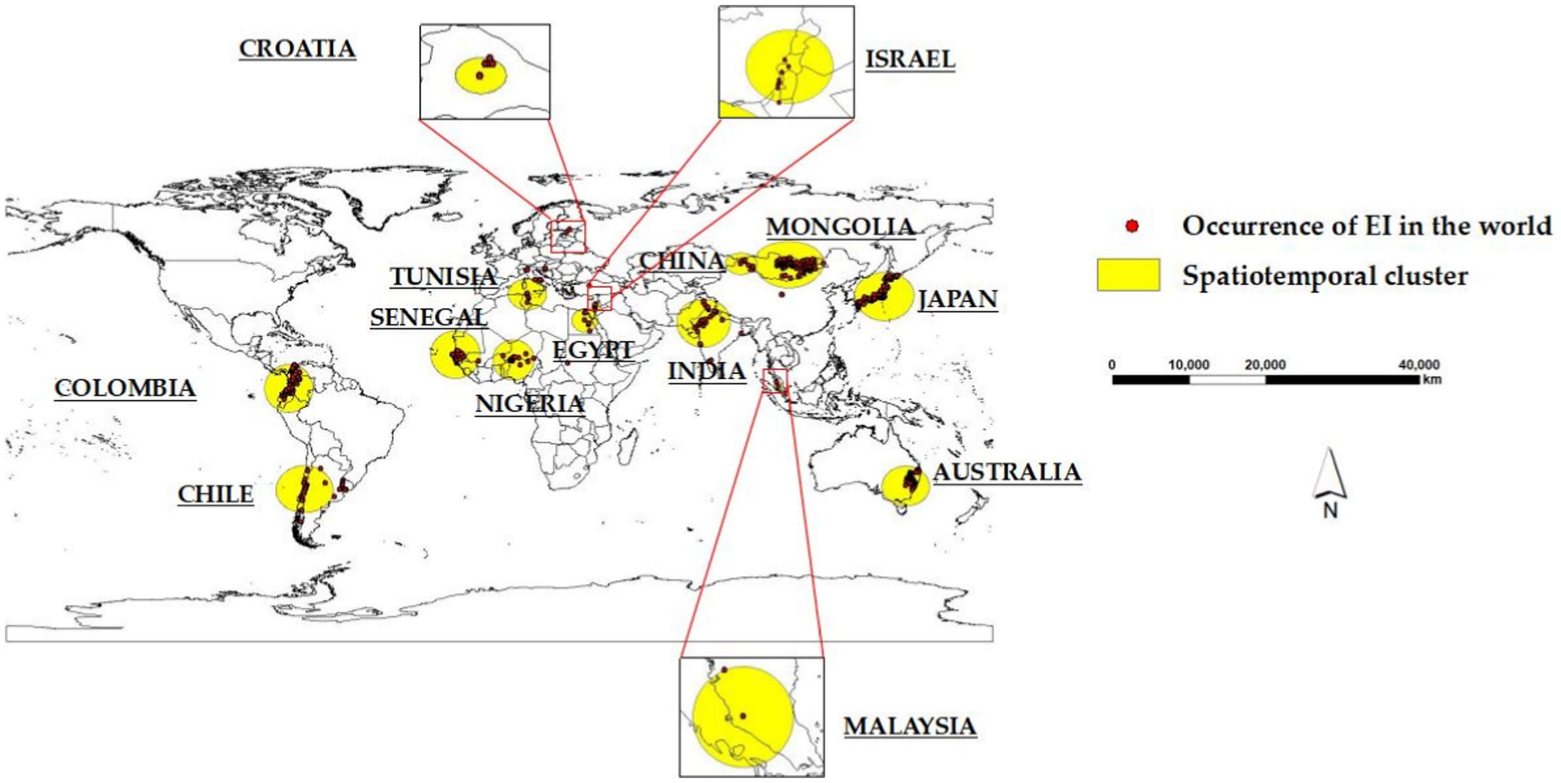
Global locations of the fourteen spatiotemporal clusters of EI occurrence.

Fourteen significant (*p* < 0.05) spatiotemporal clusters of EI occurrence were identified, along with their location coordinates, country names, radii, times, GLRs, and *p* values, as shown in Table 2. The central countries of the fourteen spatiotemporal clusters were located in Senegal, Mongolia, India, China, Colombia, Japan, Nigeria, Australia, Chile, Tunisia, Egypt, Israel, Croatia, and Malaysia, respectively. The global locations of the fourteen spatiotemporal clusters of EI occurrence were shown in Figure 3.

**Table 2:** Fourteen global spatiotemporal clusters of EI occurrence from the year 2005 to 2022.

Cluster	Center coordinates	Country	Radius (km)	Time	GLR	*p*_value
1	(14.75 N, 16.11 W)	Senegal	965.91	2019	27816.06	<0.001
2	(47.61 N, 105.54E)	Mongolia	937.93	2011	26687.18	<0.001
3	(26.26 N, 74.38E)	India	977.75	2008	23945.40	<0.001
4	(47.87 N, 88.06E)	China	432.23	2007	23430.14	<0.001
5	(2.41 N, 76.90 W)	Colombia	975.01	2018	10073.61	<0.001
6	(35.95 N, 140.32E)	Japan	976.05	2007	2421.48	<0.001
7	(12.77 N, 5.03E)	Nigeria	821.41	2018	1714.02	<0.001
8	(33.13S, 148.17E)	Australia	810.09	2007	1505.60	<0.001
9	(34.27S, 71.22 W)	Chile	955.92	2012	1242.46	<0.001
10	(36.79 N, 10.10E)	Tunisia	628.36	2020	1003.37	<0.001
11	(27.21 N, 31.11E)	Egypt	469.10	2008	755.85	<0.001
12	(32.74 N, 35.27E)	Israel	171.11	2017	516.30	<0.001
13	(45.74 N, 16.61E)	Croatia	32.75	2015	91.74	<0.001
14	(3.05 N, 101.71E)	Malaysia	184.09	2015	33.36	<0.001

### 3.2 Maxent modeling

**Figure 4:**
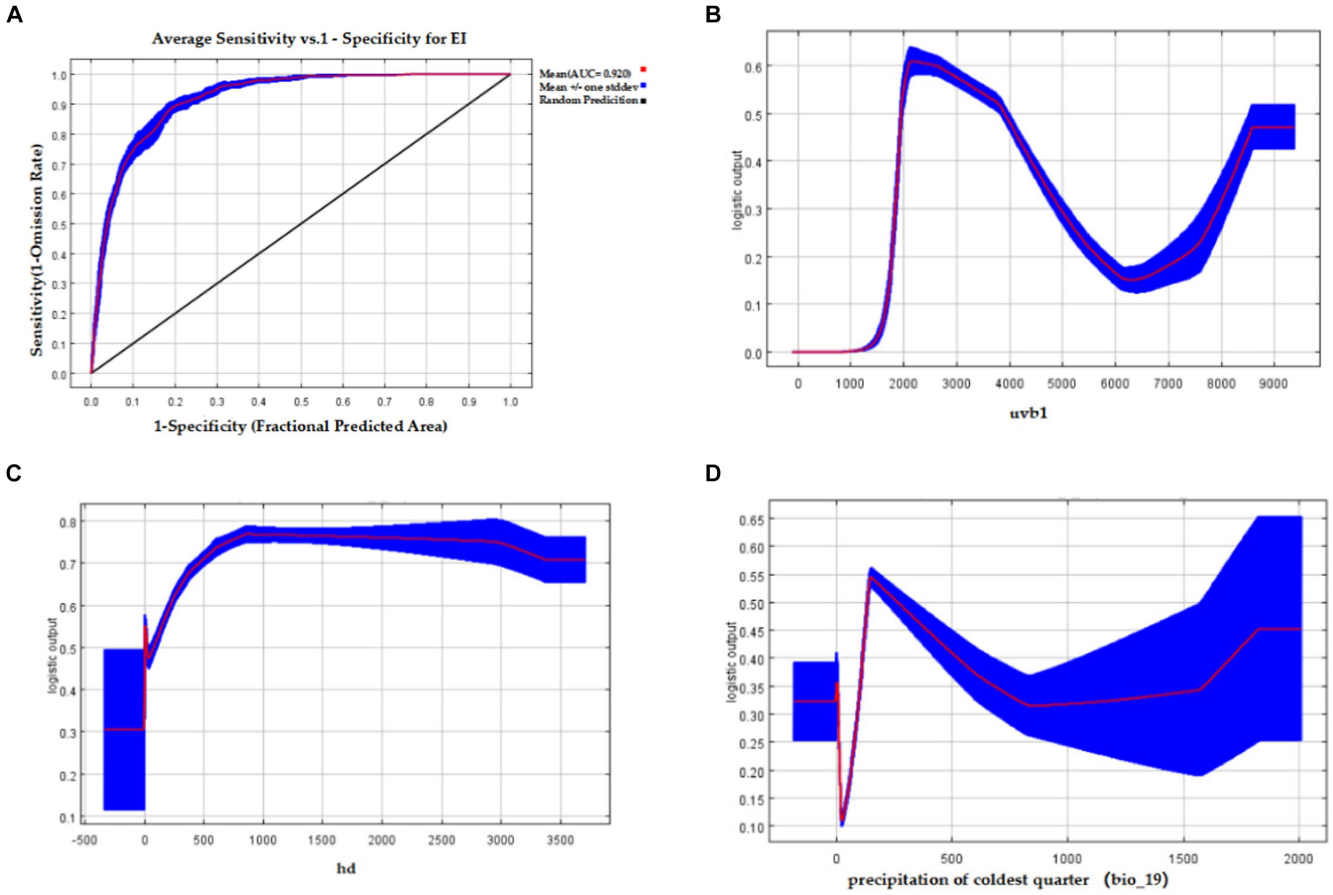
The AUC of the Maxent model for EI occurrence and response curves of three important environmental variables affecting EI occurrence. **(A)** The AUC value of the Maxent model of EI occurrence; **(B)** Response curve of annual average ultraviolet radiation (uvb1) affecting EI occurrence; **(C)** Response curve of horse density (hd) affecting EI occurrence; **(D)** Response curve of precipitation of coldest quarter (bio_19) affecting EI occurrence.

The Maxent model achieved an AUC value of 0.920 ± 0.008 for EI occurrence (Figure 4A). The environmental variables with the highest contribution rates to EI occurrence were uvb1 (32%), hd (28.2%), and bio_19 (14.5%). These three variables had a cumulative contribution rate of 74.7%. According to the response curves, the probability of EI occurrence was highest at approximately 2000 J/m^2^/day of uvb1 (Figure 4B). Moreover, as the range of hd increased from 0–800 bolt/km^2^, the probability of EI occurrence also increased until it reached 800 bolt/km^2^, beyond which it remained stable (Figure 4C). Additionally, the highest probability of EI occurrence was observed when bio_19 reached 150 mm (Figure 4D).

**Figure 5:**
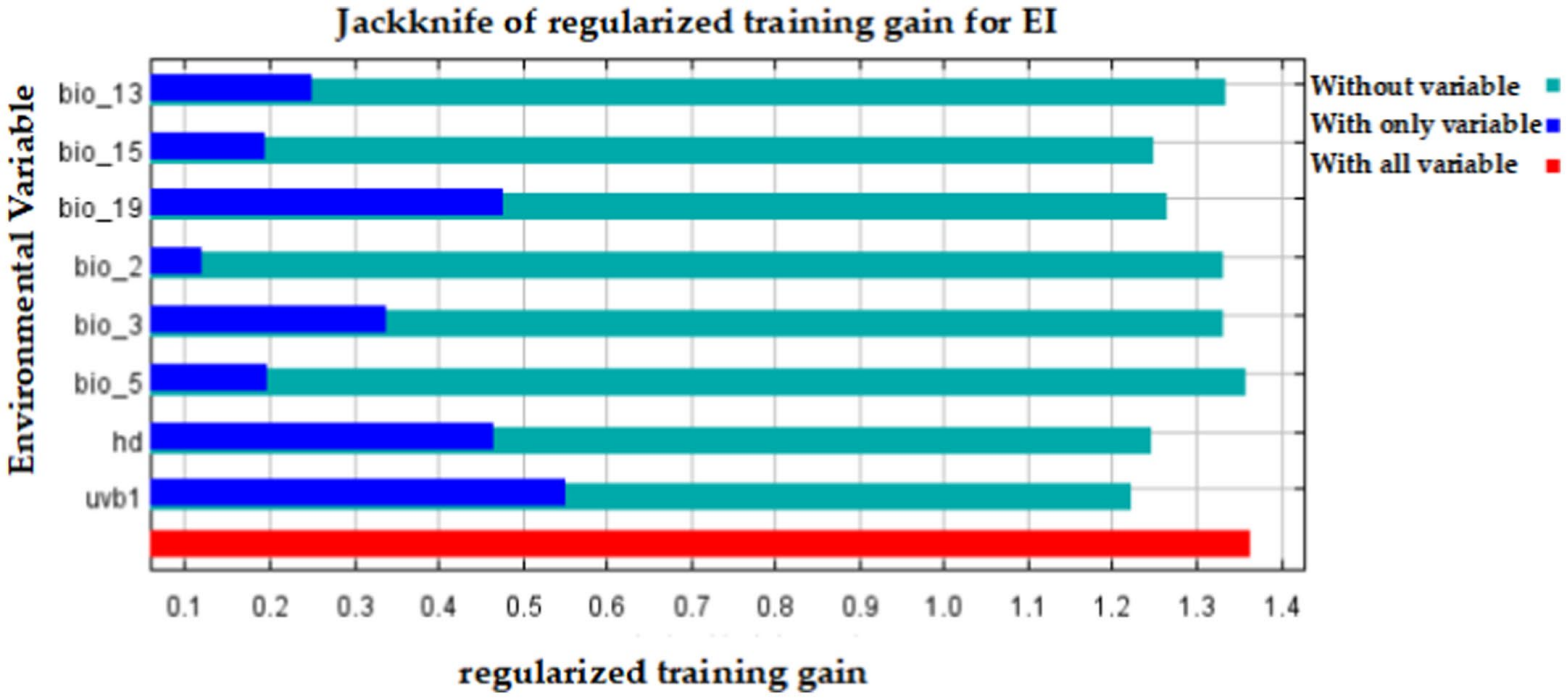
The importance analysis of eight environmental variables for EI occurrence in the Maxent model by the Jackknife test.

The importance analysis of eight environmental variables for EI occurrence in the Maxent model by the Jackknife test was shown in Figure 5. The model’s performance is represented by the red stripe when all variables are used, the dark blue stripe when only one variable is used, and the light blue stripe when only one variable is removed. The results showed that the use of solely environmental variables led to the highest gain for uvb1, followed by hd and bio_19. When only one variable was removed, uvb1 had the greatest impact, followed by hd and precipitation seasonality (bio_15), as shown in Figure 5.

**Figure 6:**
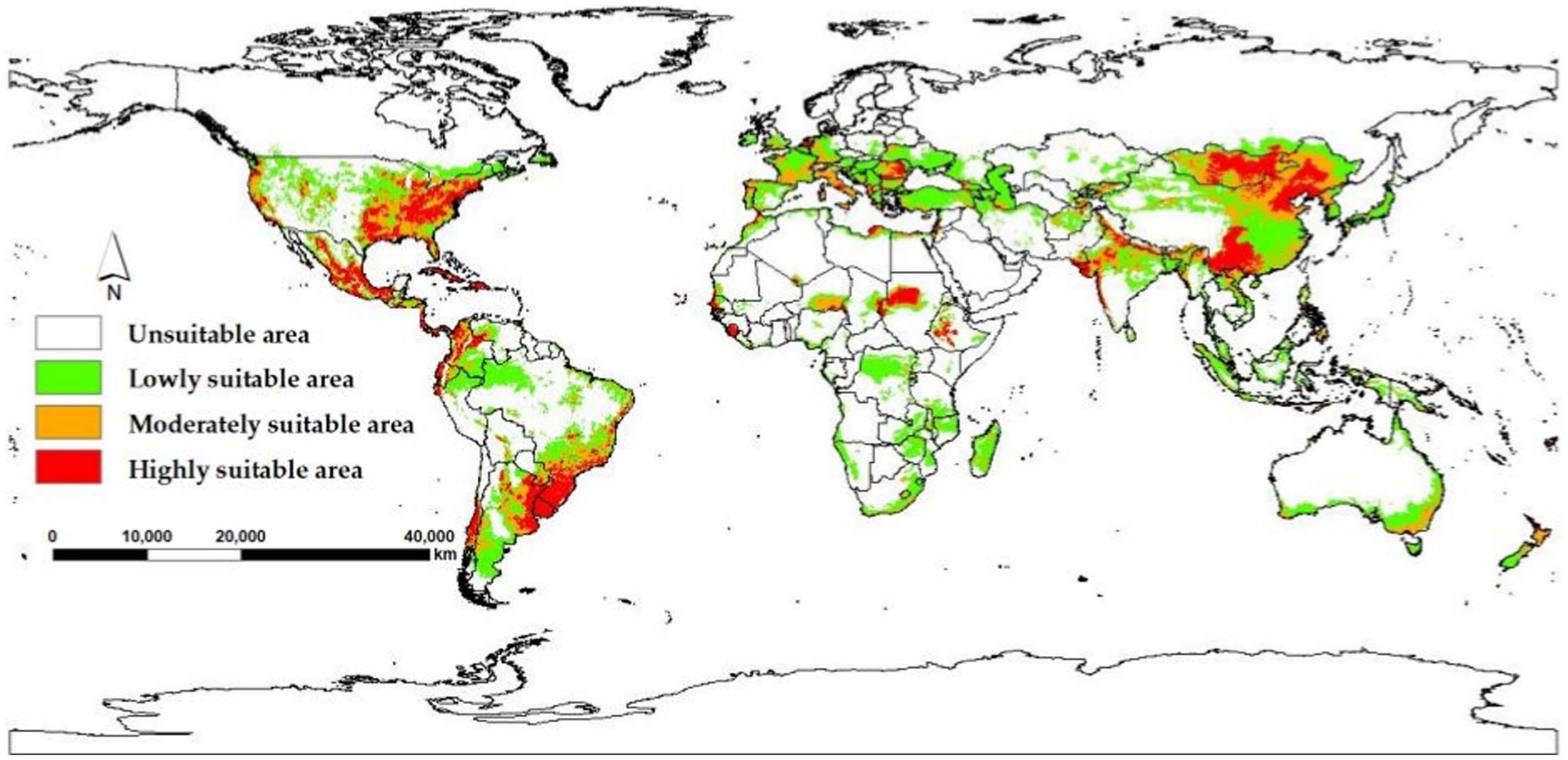
Global suitable areas of EI occurrence under the current climate scenario.

**Figure 7:**
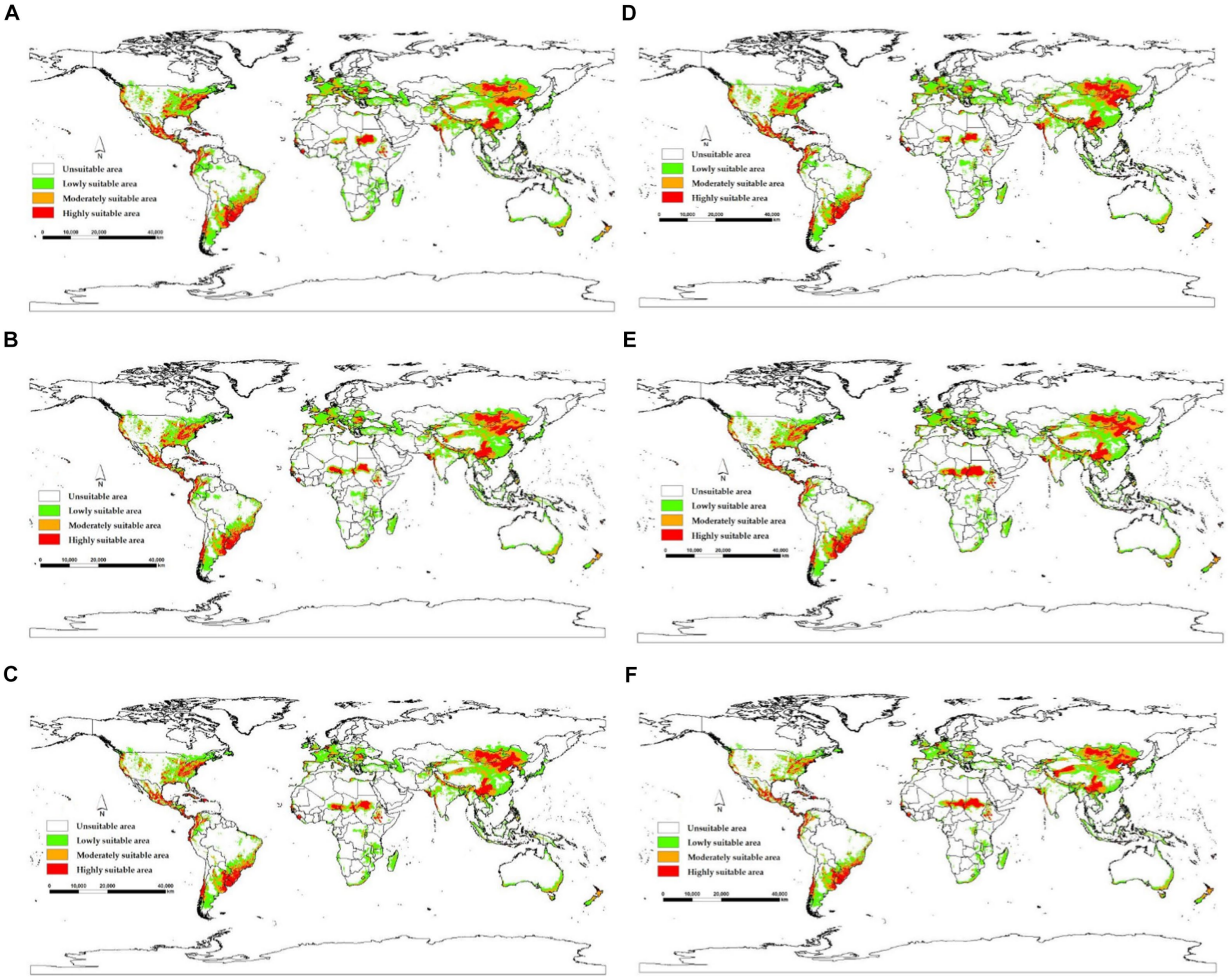
Global suitable areas of EI occurrence under three future climate scenarios for the years 2050 and 2070. **(A)** 2050SSP126, **(B)** 2050SSP245, **(C)** 2050SSP585, **(D)** 2070SSP126, **(E)** 2070SSP245, **(F)** 2070SSP585.

**Figure 8:**
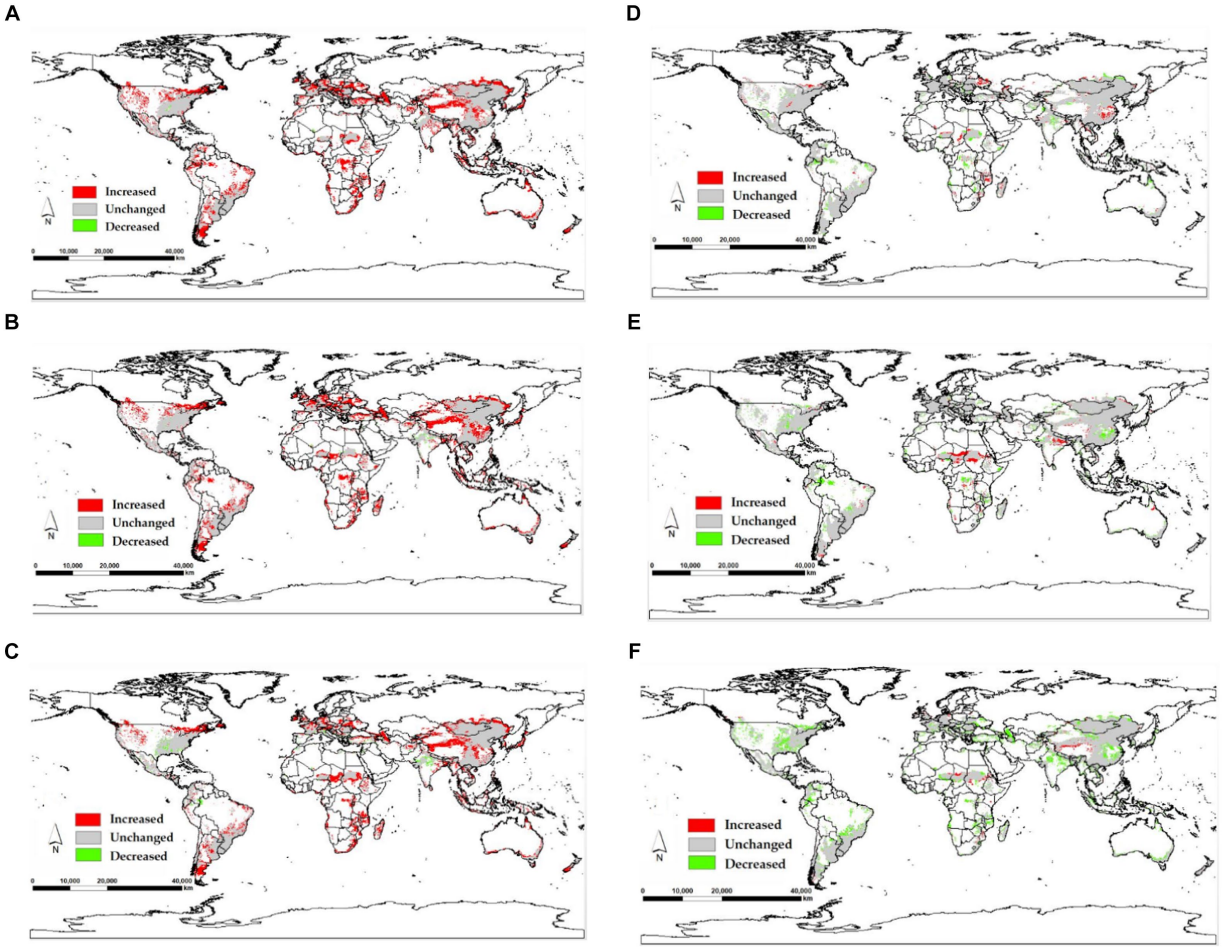
Changes in global suitable areas of EI occurrence under three future climate scenarios for the years 2050 and 2070. **(A)** 2050SSP126, **(B)** 2050SSP245, **(C)** 2050SSP585, **(D)** 2070SSP126, **(E)** 2070SSP245, **(F)** 2070SSP585.

The model yields the MTSPS value of 0.4, the division of the suitable areas of EI occurrence: unsuitable (0–0.4), lowly suitable (0.4–0.5), mediumly suitable (0.5–0.6), and highly suitable (0.6–1). The global suitable areas of EI occurrence under the current and future climate scenarios of SSP126, SSP245, and SSP585 pathways for years 2050 and 2070 are shown in Figures 6, 7, respectively. Figure 6 shows that the suitable areas were globally distributed, with significant concentrations in Asia (India, Mongolia, and China), Europe (Czech Republic), Africa (Sudan), South America (Brazil and Uruguay), and North America (USA and Mexico) under the current climate scenario. In Figure 7, the suitable areas will show an overall expansion trend, particularly in Asia and the Americas, under the future climate scenarios. Additionally, changes in global suitable areas of EI occurrence under three future climate scenarios for the years 2050 and 2070 were shown in Figure 8 and Table 3. Notably, by 2050, the suitable areas will obviously expand, with the area of the SSP126 scenario increasing by nearly 145.42 × 10^5^ km^2^. By 2070, the expansion of suitable areas will no longer be obvious, with SSP585 scenario’s area even shrinking by 59.67 × 10^5^ km^2^.

**Table 3:** Changed area in global suitable areas of EI occurrence under the SSP126/SSP245/SSP558 climate scenarios for the years 2050 and 2070.

Climate scenario	2050 (Area (× 10^5^ km^2^))			2070 (Area (× 10^5^ km^2^))		
	Increased	Unchanged	Decreased	Increased	Unchanged	Decreased
SSP126	145.42	244.62	0.61	16.03	369.11	20.93
SSP245	132.01	243.69	1.53	16.24	348.81	26.89
SSP585	113.74	238.38	6.84	7.66	292.45	59.67

**Figure 9:**
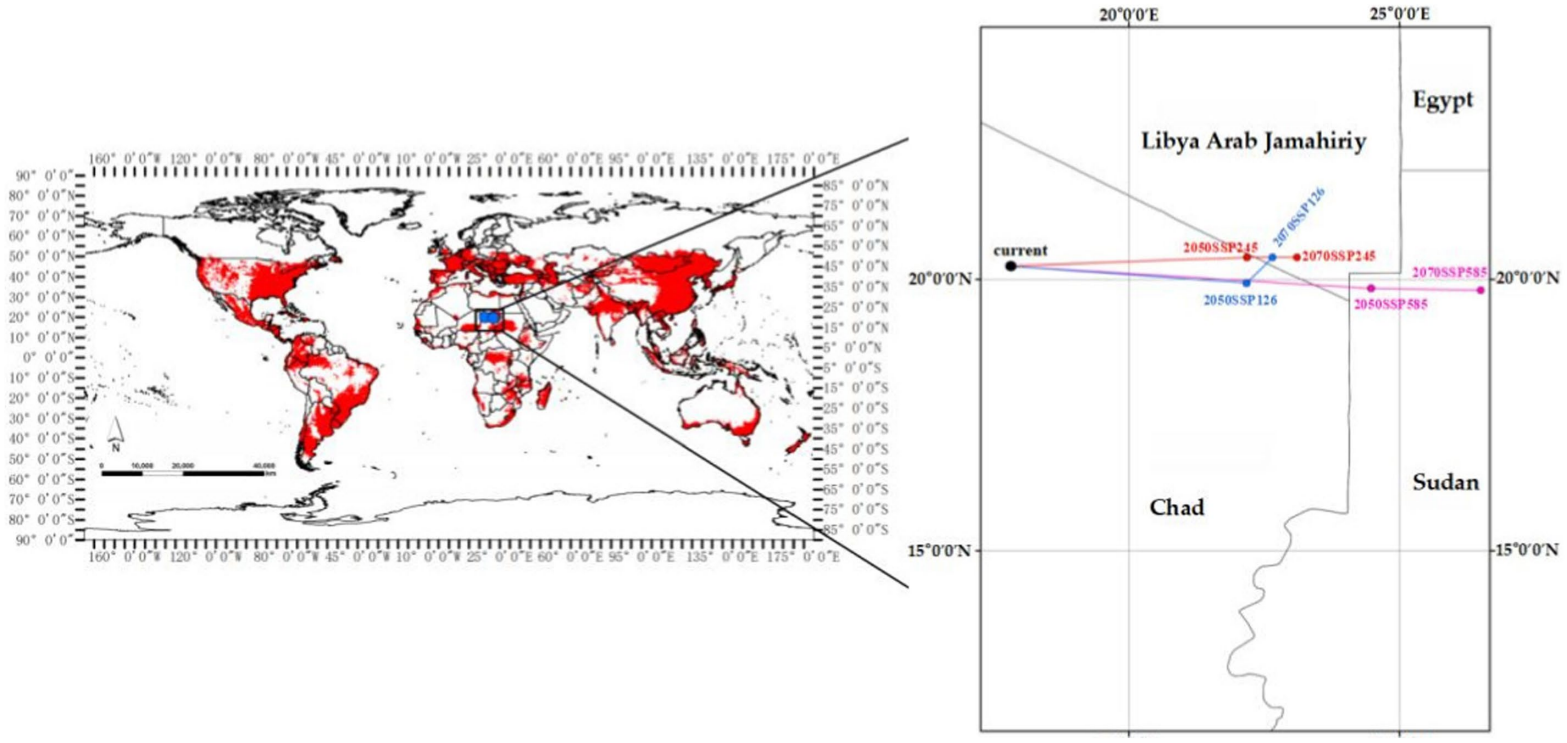
Centroid position of EI occurrence shifts under three climate scenarios from current to the years 2050 and 2070 (The blue, red and purple lines, respectively, represent the SSP126, SSP245, and SSP585 climate scenarios).

The centroid position of EI occurrence shifts from left to right under the SSP126/SSP245/SSP558 climate scenarios from current to the years 2050 and 2070, as shown in Figure 9. The current centroid position is located in Chad at geographic coordinates of (17°80′E, 20°24′N). Under the SSP126 climate scenario, the centroid will shift 417 km eastward to the position (22°17′E, 19°93′N) in 2050, and shift 104 km northeast to the position (22°65′E, 20°40′N) in 2070. Specifically, under the SSP245 climate scenario, the centroid will shift 626 km eastward to position (22°19′E, 20°40′N) in 2050, and shift nearly 313 km eastward to position (23°11′E, 20°41′N) in 2070. Under the SSP585 climate scenario, the centroid will continuously shift eastward from 2050 (24°48′E, 19°83′N) to 2070 (26°50′E, 19°80′N). Overall, the centroid position will shift eastward to varying degrees under three climate scenarios.

## 4 Discussion

EI is an important infectious disease in the global horse industry (29). Understanding the epidemiological characteristics of EI is crucial for its effective prevention and control. Previous literature has demonstrated that environmental variables such as temperature, humidity, and the number of susceptible animals play a crucial role in the transmission of the influenza virus (30, 31). Although scientists have made efforts, the environmental variables associated with EI occurrence and their roles are still unclear. This study is the first to utilize spatial epidemiological technologies to analyze the epidemiological characteristics of EI, including spatiotemporal cluster distribution, important environmental variables, and suitable areas for occurrence. The aim is to effectively improve the EI’s prevention and control effect.

This study identified fourteen spatiotemporal clusters of EI occurrence. EI was found to occur in almost all years between 2005 and 2022, and was widely distributed across all continents. The majority of spatiotemporal clusters were found in Asia (6/14), with early onset and long duration. Four spatiotemporal clusters were subsequently found in Africa, with almost all of them concentrated between 2017 and 2022. Previous studies have also reported a high incidence of EI in these four spatiotemporal clusters (32). These results suggest that EI is currently prevalent in Africa, and related countries should actively work on prevention and control measures. The remaining spatiotemporal clusters were located in the Americas, Europe, and Oceania. The results indicated that EI spatiotemporal clusters were widely distributed. The import and export of horses, as well as transnational horse competitions, pose a challenge to EI prevention and control (33). Further exploration of the risk of EI being affected in these situations is warranted.

The Maxent model had an AUC value of 0.920 ± 0.008, indicating successful establishment (25). The results suggest that annual mean ultraviolet radiation (uvb1) is a significant variable affecting the occurrence of EI. Although no study has clarified how ultraviolet radiation affects the occurrence of EI, it has been reported that the similar influenza virus is sensitive to ultraviolet radiation (34). To improve prevention and control of EI, it may be beneficial to conduct more specific studies on how ultraviolet radiation affects its occurrence. In addition, horse density (hd) is a key variable, as higher densities result in the clustering of more susceptible animals, and combined with the high incidence of EI and its rapid rate of spread, an increase in horse density can greatly contribute to the spread of EI (35). At present, spatial epidemiological studies of several infectious diseases also show that ultraviolet light and animal density play an important role in the occurrence and spread of infectious diseases (11, 36), and more precise and detailed effects need to be supported by more data from direct experiments or other epidemiological studies. Another important variable for EI occurrence is bio_19, which measures the precipitation during the coldest quarter. Firestone et al. reported that the spread of EI could be affected by relative humidity and temperature (16). As horses are homeothermic animals, they take steps to avoid damage caused by low temperatures when the environmental temperature drops to the critical lower limit of the body temperature (37). These steps include hiding in human-built shelters or congregating, which may increase the risk of direct transmission (38).

The global suitable areas for EI occurrence were predicted under the current and future climate scenarios. Under the current climate scenario, the suitable areas were mainly concentrated in Asia and the Americas, consistent with reported epidemic locations (39). Three future climate scenarios (SSP126, SSP245, and SSP585) demonstrate the impact of human activities on climate under different socioeconomic development paths. Under the future climate scenarios, the suitable areas are projected to be larger in 2050 than in 2070. The SSP126 scenario showed the largest increase in suitable areas, while the SSP585 scenario showed the largest reduction. This could be due to the fact that the SSP126 scenario had the least carbon emissions and the slowest rise in temperature, whereas the SSP585 scenario had the opposite (21). These results support the findings of the study that low temperature is a significant environmental factor affecting the occurrence of EI. It is recommended to intensively monitor the suitable areas based on the indications from future climate scenarios, particularly SSP126. The centroid position of suitable areas is expected to shift eastward in the future, indicating that there may be more suitable areas to the east of the centroid. It is reasonable to assume that these areas have favorable environmental conditions and a sufficient number of susceptible animals, creating conditions for the occurrence of EI. Once an EI occurs in these areas, the EIV can persist for an extended period, and the risk of an epidemic remains (40).

Indeed, this study has unavoidable limitations, including underreporting and time lags due to data collected from official reports. The Maxent model works in such a way that the model can achieve good modeling results even when some data is missing (41), so the limitations due to missing data are limited. Due to time and technical level, we did not apply extrapolation detection (ExDet) to evaluate model performance and identify potential overfits (42), which we will work on in future studies. Trade and transportation are critical human factors that may influence the occurrence of EI (33), these factors also contribute to the epidemic outbreak of the disease, which are not considered in this study, because accurate and comprehensive data information cannot be obtained.

With the rise in international trade and commercial activities such as exhibitions and competitions, the risk of EI occurrence is expected to increase. Therefore, effective prevention and control measures for EI are necessary. In particular, focus on immunization in areas of high suitability identified in the study and pay particular attention to whether the vaccine matches the strain currently circulating in the area. Next, researchers could concentrate on developing online platforms for preventing and monitoring EI. These platforms could help analyze and visualize the global incidence of EI and provide early warnings to high-risk areas.

## 5 Conclusion

The study identified fourteen spatiotemporal clusters of EI worldwide. The important environmental variables affecting EI occurrence were annual average ultraviolet radiation, horse density, and precipitation of the coldest quarter. Suitable areas for EI are currently widely distributed across continents, particularly in Asia and the Americas. The study predicts that under future climate scenarios, the suitable areas will expand and shift eastward. These findings present the epidemiological characteristics of EI, which can serve as a valuable reference for its prevention and control.

## Data availability statement

The original contributions presented in the study are included in the article/supplementary material, further inquiries can be directed to the corresponding authors.

## Ethics statement

The manuscript presents research on animals that do not require ethical approval for their study.

## Author contributions

JD: Writing – original draft. YW: Writing – review & editing. JL: Software, Writing – review & editing. ZH: Software, Writing – review & editing. CZ: Software, Validation, Writing – review & editing. YH: Formal analysis, Writing – review & editing. JX: Investigation, Writing – review & editing. LL: Resources, Writing – review & editing. JM: Conceptualization, Writing – review & editing. MZ: Data curation, Writing – original draft. BL: Methodology, Writing – review & editing. MS: Methodology, Writing – review & editing.

## Footnotes

^1^ http://www.worldclim.org

^2^ http://www.ufz.de/gluv/

^3^ http://www.fao.org/livestock-systems/global-distributions/zh/

## References

[1] Chambers TM. Equine Influenza. Cold Spring Harb Perspect Med. (2022) 12:a038331. doi: 10.1101/cshperspect.a038331, PMID: 32152243 PMC8725621

[2] Singh RK Dhama K Karthik K Khandia R Munjal A Khurana SK. Comprehensive review on equine influenza virus: etiology, epidemiology, pathobiology, advances in developing diagnostics, vaccines, and control strategies. Front Microbiol. (2018) 9:1941. doi: 10.3389/fmicb.2018.01941, PMID: 30237788 PMC6135912

[3] Chambers TM. A brief introduction to equine influenza and equine influenza viruses In: Spackman E, editor. Animal influenza virus. methods in molecular biology. New York, NY: Springer (2014). 365–70. 10.1007/978-1-4939-0758-8_31 24899445

[4] Landolt GA. Equine influenza virus. Vet Clin North Am. (2014) 30:507–22. doi: 10.1016/j.cveq.2014.08.003 25282321

[5] Daly JM MacRae S Newton JR Wattrang E Elton DM. Equine influenza: a review of an unpredictable virus. Vet J. (2011) 189:7–14. doi: 10.1016/j.tvjl.2010.06.026, PMID: 20685140

[6] Olguin-Perglione C Barrandeguy ME. An overview of equine influenza in South America. Viruses. (2021) 13:888. doi: 10.3390/v13050888, PMID: 34065839 PMC8151294

[7] Newton JR Rendle DI Mountford DR Marr CM. Equine influenza vaccination catches an autumn cold! But must get over it as soon as it can. Equine Vet J. (2023) 55:142–6. doi: 10.1111/evj.13885, PMID: 36226994

[8] Ostfeld RS Glass GE Keesing F. Spatial epidemiology: an emerging (or re-emerging) discipline. Trends Ecol Evol. (2005) 20:328–36. doi: 10.1016/j.tree.2005.03.009, PMID: 16701389

[9] Kulldorff M Heffernan R Hartman J Assunção R Mostashari F. A space-time permutation scan statistic for disease outbreak detection. PLoS Med. (2005) 2:e59. doi: 10.1371/journal.pmed.0020059, PMID: 15719066 PMC548793

[10] Wong NS Huang SJ Chen L Zhao PZ Tucker JD Yang LG. Spatiotemporal clusters of primary and secondary syphilis cases in South China: an observational study. Lancet. (2016) 388:S90. doi: 10.1016/S0140-6736(16)32017-7

[11] Gao HY Ma J. Spatial distribution and risk areas of foot and mouth disease in mainland China. Prev Vet Med. (2021) 189:105311. doi: 10.1016/j.prevetmed.2021.105311, PMID: 33652349

[12] Deka MA Vieira AR Bower WA. Modelling the ecological niche of naturally occurring anthrax at global and circumpolar extents using an ensemble modelling framework. Transbound Emerg Dis. (2022) 69:e2563–77. doi: 10.1111/tbed.14602, PMID: 35590480 PMC10961590

[13] Warren DL Seifert SN. Ecological niche modeling in Maxent: the importance of model complexity and the performance of model selection criteria. Ecol Appl. (2011) 21:335–42. doi: 10.1890/10-1171.1, PMID: 21563566

[14] Ren H Zhao L Zhang A Song L Liao Y Lu W. Early forecasting of the potential risk zones of COVID-19 in China’s megacities. Sci Total Environ. (2020) 729:138995. doi: 10.1016/j.scitotenv.2020.138995, PMID: 32353723 PMC7252152

[15] Choi JH Namgung H Lim SJ Kim EK Oh Y Park YC. Predicting suitable areas for African swine fever outbreaks in wild boars in South Korea and their implications for managing high-risk pig farms. Animals. (2023) 13:2148. doi: 10.3390/ani13132148, PMID: 37443946 PMC10339976

[16] Firestone SM Cogger N Ward MP Toribio JA Moloney BJ Dhand NK. The influence of meteorology on the spread of influenza: survival analysis of an equine influenza (a/H3N8) outbreak. PLoS One. (2012) 7:e35284. doi: 10.1371/journal.pone.0035284, PMID: 22536366 PMC3335077

[17] Baker RE Mahmud AS Miller IF Rajeev M Rasambainarivo F Rice BL. Infectious disease in an era of global change. Nat Rev Microbiol. (2021) 9:193–205. doi: 10.3390/vetsci9110606, PMID: 34646006 PMC8513385

[18] Warren DL Glor RE Turelli M. ENMTools: a toolbox for comparative studies of environmental niche models. Ecography. (2010) 33:607–11. doi: 10.1111/j.1600-0587.2009.06142.x

[19] Poggio L Simonetti E Gimona A. Enhancing the WorldClim data set for national and regional applications. Sci Total Environ. (2018) 625:1628–43. doi: 10.1016/j.scitotenv.2017.12.258, PMID: 29996459

[20] Wu TW Lu YX Fang YJ Xin XG Li L Li WP. The Beijing climate center climate system model (BCC-CSM): the main progress from CMIP5 to CMIP6. Geosci Model Dev. (2019) 12:1573–600. doi: 10.5194/gmd-12-1573-2019

[21] Kong Y Feng C Guo L. Peaking global and G20 Countries’ CO2 emissions under the shared socio-economic pathways. Int J Environ Res Public Health. (2022) 19:11076. doi: 10.3390/ijerph191711076, PMID: 36078791 PMC9518017

[22] Liu K Sun J Liu X Li R Wang Y Lu L. Spatiotemporal patterns and determinants of dengue at county level in China from 2005–2017. Int J Infect Dis. (2018) 77:96–104. doi: 10.1016/j.ijid.2018.09.003, PMID: 30218814

[23] Guo W Li Z Liu T Feng J. Effects of climate change on the distribution of threatened fishing bat Myotis pilosus in China. Animals. (2023) 13:1784. doi: 10.3390/ani13111784, PMID: 37889742 PMC10251902

[24] Teng AY Che TL Zhang AR Zhang YY Xu Q Wang T. Mapping the viruses belonging to the order Bunyavirales in China. Infect Dis Poverty. (2022) 11:81. doi: 10.1186/s40249-022-00993-x, PMID: 35799306 PMC9264531

[25] Phillips SJ Anderson RP Schapire RE. Maximum entropy modeling of species geographic distributions. Ecol Model. (2006) 190:231–59. doi: 10.1016/j.ecolmodel.2005.03.026

[26] Liu B Jiao Z Ma J Gao X Xiao J Hayat MA. Modelling the potential distribution of arbovirus vector *Aedes aegypti* under current and future climate scenarios in Taiwan, China. Pest Manag Sci. (2019) 75:3076–83. doi: 10.1002/ps.5424, PMID: 30919547

[27] Akpan GE Adepoju KA Oladosu OR Adelabu SA. Dominant malaria vector species in Nigeria: modelling potential distribution of *Anopheles gambiae* sensu lato and its siblings with MaxEnt. PLoS One. (2018) 13:e0204233. doi: 10.1371/journal.pone.0204233, PMID: 30281634 PMC6169898

[28] Aidoo OF Souza PGC da Silva RS Santana PA Picanço MC Kyerematen R. Climate-induced range shifts of invasive species (*Diaphorina citri* Kuwayama). Pest Manag Sci. (2022) 78:2534–49. doi: 10.1002/ps.6886, PMID: 35332664

[29] Dominguez M Münstermann S de Guindos I Timoney P. Equine disease events resulting from international horse movements: systematic review and lessons learned. Equine Vet J. (2016) 48:641–53. doi: 10.1111/evj.12523, PMID: 26509734

[30] Katherine EEJ Ghedin E. Quantifying between-host transmission in influenza virus infections. Cold Spring Harb Perspect Med. (2020) 10:a038422. doi: 10.1101/cshperspect.a038422, PMID: 31871239 PMC7397841

[31] Lowen AC Mubareka S Steel J Palese P. Influenza virus transmission is dependent on relative humidity and temperature. PLoS Pathog. (2007) 3:1470–6. doi: 10.1371/journal.ppat.0030151, PMID: 17953482 PMC2034399

[32] Diallo AA Souley MM Issa Ibrahim A Alassane A Issa R Gagara H. Transboundary spread of equine influenza viruses (H3N8) in west and Central Africa: molecular characterization of identified viruses during outbreaks in Niger and Senegal, in 2019. Transbound Emerg Dis. (2021) 68:1253–62. doi: 10.1111/tbed.13779, PMID: 32770642

[33] Sack A Cullinane A Daramragchaa U Chuluunbaatar M Gonchigoo B Gray GC. Equine influenza virus—a neglected, Reemergent disease threat. Emerg Infect Dis. (2019) 25:1185–91. doi: 10.3201/eid2506.161846

[34] Juzeniene A Ma LW Kwitniewski M Polev GA Lagunova Z Dahlback A. The seasonality of pandemic and non-pandemic influenzas: the roles of solar radiation and vitamin D. Int J Infect Dis. (2010) 14:e1099–105. doi: 10.1016/j.ijid.2010.09.002 21036090

[35] Whitlock F Grewar J Newton R. An epidemiological overview of the equine influenza epidemic in Great Britain during 2019. Equine Vet J. (2023) 55:153–64. doi: 10.1111/evj.13874, PMID: 36054725 PMC10087154

[36] Jun M Hao C Xiang G Jianhua X Hongbin W. African swine fever emerging in China: distribution characteristics and high-risk areas. Prev Vet Med. (2019) 175:104863:104861. doi: 10.1016/j.prevetmed.2019.104861, PMID: 31810030

[37] Mejdell CM Bøe KE Jørgensen GHM. Caring for the horse in a cold climate—reviewing principles for thermoregulation and horse preferences. Appl Anim Behav Sci. (2020) 231:105071. doi: 10.1016/j.applanim.2020.105071

[38] Jørgensen GHM Aanensen L Mejdell CM Bøe KE. Preference for shelter and additional heat in horses exposed to Nordic winter conditions. Equine Vet J. (2016) 48:720–6. doi: 10.1111/evj.12522, PMID: 26509636

[39] OIE. World Organisation for Animal Health. Available at: https://www.woah.org/ (Accessed February 16, 2023)

[40] Gonzalez-Obando J Forero JE Zuluaga-Cabrera AM Ruiz-Saenz J. Equine influenza virus: an old known enemy in the Americas. Vaccine. (2022) 10:1718. doi: 10.3390/vaccines10101718, PMID: 36298583 PMC9610386

[41] Elith J Phillips SJ Hastie T Dudík M Chee YE Yates CJ. A statistical explanation of MaxEnt for ecologists. Divers Distrib. (2011) 17:43–57. doi: 10.1111/j.1472-4642.2010.00725.x

[42] Wang H Zhang Q Liu R Sun Y Xiao J Gao L. Impacts of changing climate on the distribution of *Solenopsis invicta* Buren in mainland China: exposed urban population distribution and suitable habitat change. Ecol Indic. (2022) 139:108944. doi: 10.1016/j.ecolind.2022.108944

